# Age- and season-dependent pattern of flavonol glycosides in Cabernet Sauvignon grapevine leaves

**DOI:** 10.1038/s41598-020-70706-7

**Published:** 2020-08-28

**Authors:** Sakina Bouderias, Péter Teszlák, Gábor Jakab, László Kőrösi

**Affiliations:** 1 Research Institute for Viticulture and Oenology, University of Pécs, Pázmány P. u. 4, Pécs, 7634 Hungary; 2 Department of Plant Biology, University of Pécs, Ifjúság u. 6, Pécs, 7624 Hungary

**Keywords:** Biochemistry, Plant sciences

## Abstract

Flavonols play key roles in many plant defense mechanisms, consequently they are frequently investigated as stress sensitive factors in relation to several oxidative processes. It is well known that grapevine (*Vitis vinifera* L.) can synthesize various flavonol glycosides in the leaves, however, very little information is available regarding their distribution along the cane at different leaf levels. In this work, taking into consideration of leaf position, the main flavonol glycosides of a red grapevine cultivar (Cabernet Sauvignon) were profiled and quantified by HPLC–DAD analysis. It was found that amount of four flavonol glycosides, namely, quercetin-3-*O*-galactoside, quercetin-3-*O*-glucoside, kaempferol-3-*O*-glucoside and kaempferol-3-*O*-glucuronide decreased towards the shoot tip. Since leaf age also decreases towards the shoot tip, the obtained results suggest that these compounds continuously formed by leaf aging, resulting in their accumulation in the older leaves. In contrast, quercetin-3-*O*-glucuronide (predominant form) and quercetin-3-*O*-rutinoside were not accumulated significantly by aging. We also pointed out that grapevine boosted the flavonol biosynthesis in September, and flavonol profile differed significantly in the two seasons. Our results contribute to the better understanding of the role of flavonols in the antioxidant defense system of grapevine.

## Introduction

Flavonoids are very important secondary metabolites, having various functional roles in different physiological and developmental processes in plants^1–3^. Flavonols as a group of flavonoids are mainly accumulated in epidermal cells of plant tissues in response to solar radiation^4, 5^, to filter the UV-B light while allowing to pass the photosynthetically active visible light^6–8^. Besides their photoprotective roles^9, 10^, flavonols have an antioxidant function during plant response to different environmental stress^5, 10, 11^. Because of their high antioxidant capacity, the nutritional and beneficial health effects of these compounds have been extensively studied in the recent years^12–14^.

Grapevine (*Vitis vinifera* L.) cultivars are rich in polyphenols, especially flavonol derivatives such as quercetin- and kaempferol glycosides^15–18^. The amount and composition of phenolics vary in different organs. Higher flavonol contents were found both leaves and petioles compared to the berries^19, 20^. The level of these compounds depends on the grape variety as well as both biotic and abiotic stresses. In stressed plants, the level of reactive oxygen species (ROS) is outbalanced over the antioxidant compounds. Stressors can induce the activation of the defense system, which increases the biosynthesis of many phenolic compounds. ESCA disease induced differential phenolic production in *V. vinifera* leaves^21^. Flavonoids and hydroxycinnamoyl tartaric acids were strongly correlated with disease. *Plasmopara viticola* infection also led to the production and accumulation of stress metabolites such as flavonoids, phenylpropanoids and some amino acids in a resistant grapevine, ‘Regent’, leaves^22^. Phytoplasma infection (*Bois noir*) led to an increase of the contents of hydroxycinnamic acids, flavanols and flavonols (quercetin-3-*O*-glucuronide, quercetin-3-*O*-glucoside)^23^. Strong photocatalytic stress using TiO_2_ nanoparticles decreased the level of quercetin-3-*O*-glucuronide in Cabernet Sauvignon, Cabernet Franc and Kékfrankos leaves while boosted the biosynthesis of other flavonols on genotype dependent manner^15^. Long-term drought stress caused a decrease in selected elements of secondary metabolism not only in the leaves but also in roots of the grapevine^24^. Similar to long-term drought, prolonged cold stress induced the reduction of phenolic acids in both susceptible and more-tolerant grapevine varieties^25^. Quercetin-3-*O*-glucoside and kaempferol-3-*O*-glucoside found to be UV-B stress indicators in the leaves of *V. vinifera* cultivars Pinot Noir and Riesling^26^. Therefore, leaf polyphenols, especially flavonol content can be used as an indicator of stress history of the plant^27^.

Although the nature of the flavonols in the grapevine leaves are well-documented, the fluctuation of their level along the shoot is scarcely investigated. It was reported that the concentration of kaempferol-3-*O*-glucoside and its aglycon differed significantly depending on the leaf age in Pinot Noir cultivar^28^. However, in the previous work, leaves only with three age groups collected in one season were investigated in greenhouse conditions^28^. Thus, there is no detailed information about flavonol pattern as a function of leaf ages.

In this work, flavonol glycosides of a red grapevine cultivar, *Vitis vinifera* L. cv. Cabernet Sauvignon, were analyzed in detail by means of high-performance liquid chromatography, taking into account the individual leaf levels under field conditions. The purpose of our work to reveal the distribution of the main flavonols in the leaves considering the leaf position and the harvesting season. Profiling these stress-sensitive flavonols is crucial for stress biological studies and gives beneficial information for proper sampling. In the absence of these basic information, the flavonol responses of stressed plants can be misinterpreted or lead to incorrect conclusions.

## Results

### The impact of the leaf position on flavonol glycoside patterns of Cabernet Sauvignon leaves

**Figure 1:**
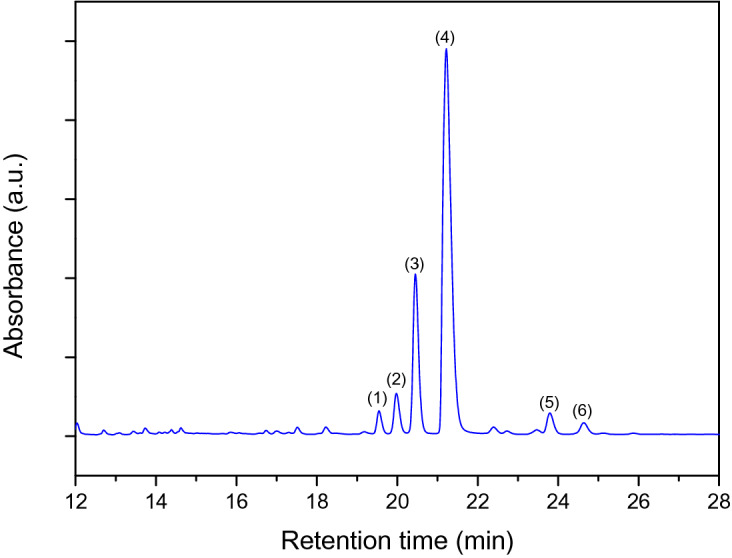
Typical HPLC–DAD chromatogram of methanolic leaf extract of Cabernet Sauvignon: (**1**) quercetin-3-*O*-rutinoside, (**2**) quercetin-3-*O*-galactoside, (**3**) quercetin-3-*O*-glucoside, (**4**) quercetin-3-*O*-glucuronide, (**5**) kaempferol-3-*O*-glucoside and (**6**) kaempferol-3-*O*-glucuronide.

A typical HPLC–DAD chromatogram of methanolic leaf extract of Cabernet Sauvignon was presented in Fig. 1. The identified key flavonols, quercetin and kaempferol, were present in various glycosylated forms. Six flavonol glycosides, namely, quercetin-3-*O*-rutinoside (Q-rut), quercetin-3-*O*-galactoside, (Q-gal), quercetin-3-*O*-glucoside (Q-glc), quercetin-3-*O*-glucuronide (Q-glr), kaempferol-3-*O*-glucoside (K-glc) and kaempferol-3-*O*-glucuronide (K-glr) were identified. Q-glr was the predominant derivative with high concentration (~ 12,650–16,520 ppm DW) while the second most abundant flavonol glycoside was Q-glc (~ 1,125–4,380 ppm DW). Q-rut, Q-gal and kaempferol glycosides were detected in lower concentration range, typically below ~ 1,000 ppm DW. The chemical structures of these phenolics are displayed in Figure S1.

**Figure 2:**
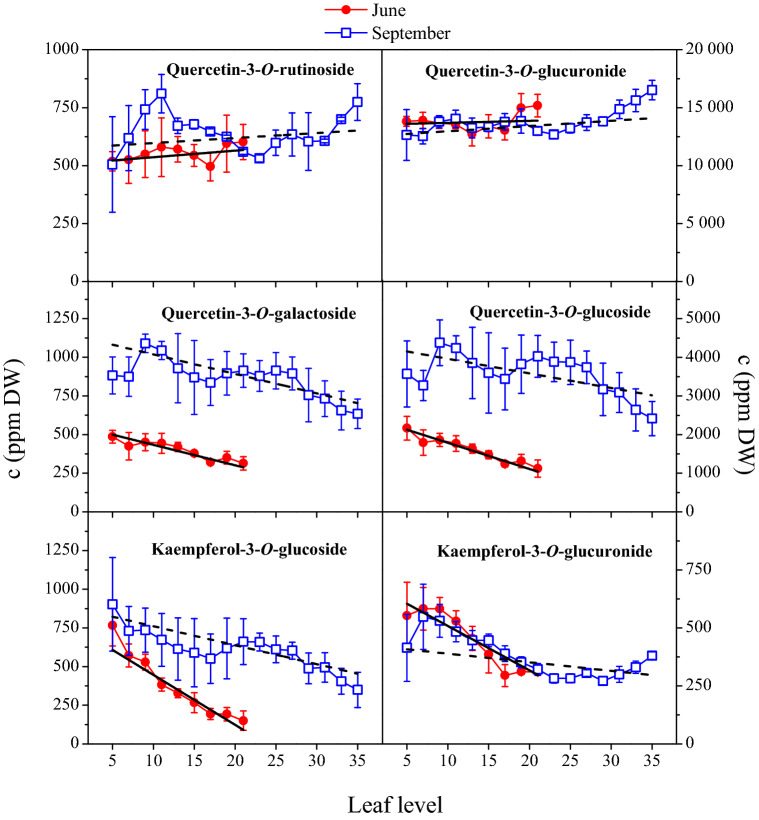
Concentration changes of various flavonol glycosides in Cabernet Sauvignon leaves as a function of leaf levels. Concentrations are expressed in μg g^−1^ dry weight. Linear fitting marked with solid or dashed lines was performed by Origin 8.6.0 software.

Figure 2 shows how the concentration of the individual flavonol glycosides changed as a function of leaf level. Based on these variations, the six flavonol glycosides can be divided into two groups. The first group includes Q-gal, Q-glc, K-glc and K-glr. The concentrations of these flavonol derivatives showed a decreasing trends towards the shoot tip, resulting that younger leaves contained lower amount of them (Tables S1 and S2). Q-rut and Q-glr can be classified into the second group. Their concentrations were not proved to be significantly lower in the younger leaves (Fig. 2). Indeed, concentrations of Q-rut and Q-glr slightly increased, especially for the last leaf levels. The changes of the flavonol levels are well indicated by the slope of the lines fitted to the data points (Fig. 2). In June, for the Q-rut and Q-glr contents, there was no significant difference between old and young leaves (Table S1). In September, Q-glr level was significantly higher in younger leaves (Table S2).

Statistical analysis also revealed another interesting relation. Strong positive correlations were found between the Q-glr and Q-rut levels, while Q-glc content positively correlated with Q-gal, and K-glc contents. The Pearson’s correlation coefficients are listed in Table 1.

**Table 1:** Pearson’s correlation analysis between flavonol glycosides (Q-rut, Q-gal, Q-glc, Q-glr, K-glc, K-glr) and total flavonols (TF) of Cabernet Sauvignon leaves collected in June and September. Pearson's correlation coefficients were calculated pair wise for n = 27 and n = 48 data sets in June and September, respectively.

	Q-rut	Q-gal	Q-glc	Q-glr	K-glc	K-glr	TF
**June**							
Q-rut	1	0.248	0.002	0.406*	− 0.249	− 0.099	0.393*
Q-gal		1	0.961**	− 0.178	0.751**	0.719**	0.437*
Q-glc			1	− 0.207	0.864**	0.741**	0.426*
Q-glr				1	− 0.195	− 0.246	0.786**
K-glc					1	0.843**	0.399*
K-glr						1	0.314
TF							1
**September**							
Q-rut	1	0.215	0.122	0.614**	− 0.067	0.535**	0.685**
Q-gal		1	0.964**	− 0.253	0.797**	0.417**	0.505**
Q-glc			1	− 0.23	0.800**	0.262	0.521**
Q-glr				1	− 0.304*	0.064	0.697**
K-glc					1	0.449**	0.368*
K-glr						1	0.381**
TF							1

*Correlation is significant at the 0.05 level.

**Correlation is significant at the 0.01 level.

**Figure 3:**
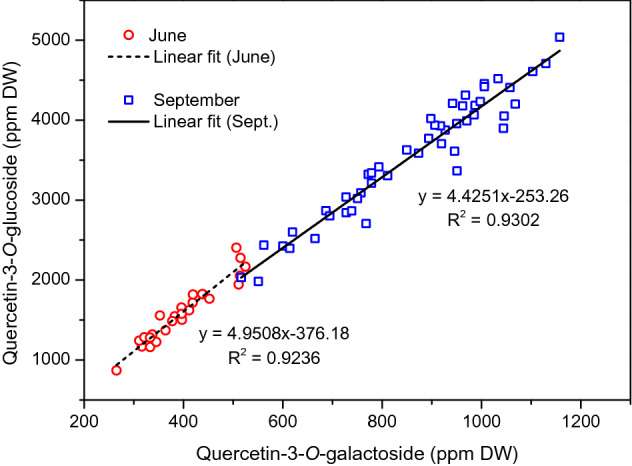
Plotting of quercetin-3-*O*-glucoside as a function of quercetin-3-*O*-galactoside in June (hollow circles) and September (hollow squares). Fitted lines are marked with dashed or solid line. The results of linear regression are displayed below the corresponding data.

The strongest correlation (r = 0.961) was found between Q-gal and Q-glc. By plotting of Q-glc as a function of Q-gal, the linear fit resulted in R^2^ > 0.92, showing that the relationship between the two components is nearly linear (Fig. 3). The slopes of the fitted lines are similar in June and September, indicating comparable Q-glc:Q-gal ratios in both seasons. The data points in the two seasons were not overlapped, but they appeared in two separated ranges, closely following each other. This clearly shows that all the leaves (including the youngest ones) had higher Q-gal and Q-glc levels in September than in June. In June, weaker but significant positive correlations were found between Q-gal and K-glc; Q-gal and K-glr; finally, K-glc and K-glr levels (Table 1). For these data pairs, the slopes of the fitted lines differed in the two seasons, indicating their different proportions in June and September (Figure S2). There was also a positive correlation between Q-rut and Q-glr levels (Table 1, Figure S2). However, the slope of the fitted lines was very similar showing the same Q-rut:Q-glr ratio in both seasons. In September, there were strong positive correlations between Q-rut, Q-gal, K-glc and K-glr.

### Seasonal variation of the flavonol content in Cabernet Sauvignon leaves

Figure 2 also demonstrates that grapevine accumulated Q-gal, Q-glc, and K-glc in September. By comparing the leaf compositions in different seasons, the individual flavonol glycosides showed concentration changes with similar patterns along the shoots up to 22th leaf level (Fig. 2). The similarity of the concentration changes in the two seasons were remarkable for all flavonol glycosides. Even though the amount of Q-gal and Q-glc was multiplied until September, the trends in their concentration changes towards the shoot tip were similar in both seasons. Student’s t-tests confirmed that autumn leaves contained significantly higher level of Q-gal, Q-glc, K-glc and K-glr (Table S3).

**Figure 4:**
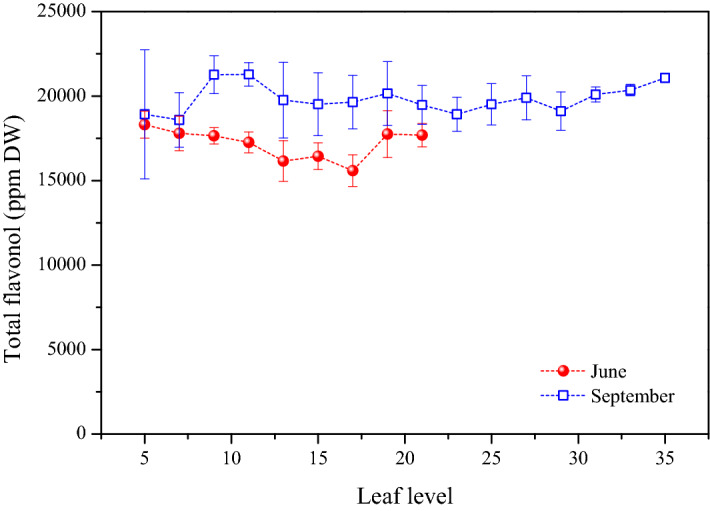
Total flavonol level of Cabernet Sauvignon leaves harvested in June and September as a function of leaf levels.

The level of total flavonols (adding together the level of individual FLGs) were proved to be higher in September independently from the leaf position (Fig. 4). Student’s t-tests showed that these differences were significant (Tables S3 and S4). Although the season influenced the total flavonol level, there were no significant differences between the total flavonol content of the young and old leaves in one season (Tables S1 and S2).

**Figure 5:**
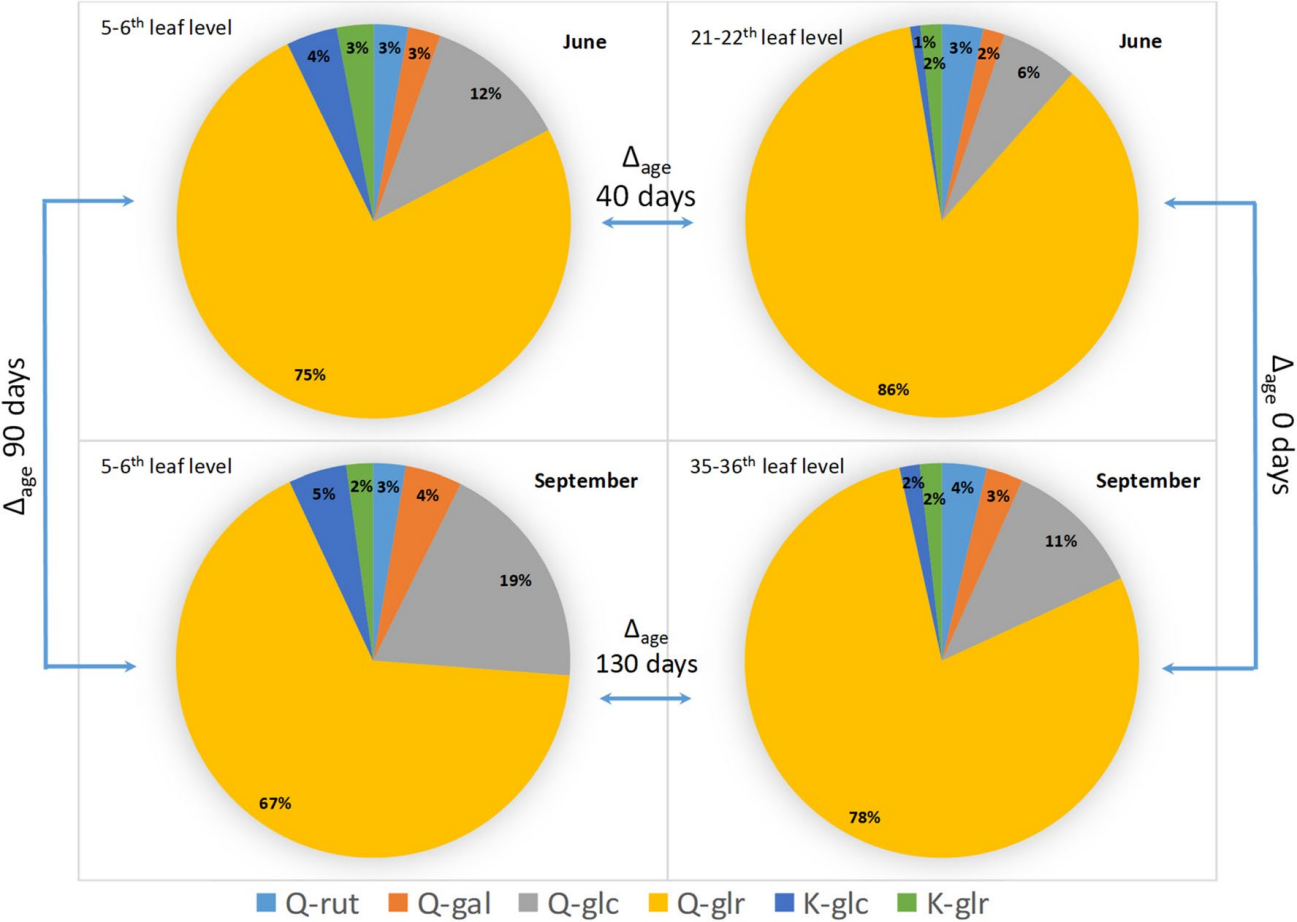
Flavonol profiles of the oldest and the youngest Cabernet Sauvignon leaves harvested in June and September.

The flavonol profile of youngest and the oldest leaves in both seasons are compared in Fig. 5. In June, the Q-glr ratio (calculated by level of Q-glr per level of total flavonols) for the youngest leaves (21–22th levels) was 86% while it was 75% for the oldest ones (5–6th levels). In this comparison, the difference between the leaf ages is 40 days. While Q-glr ratio decreased by the leaf ageing, Q-glc fraction increased from 6 to 12%. At same time K-glc fraction also increased from 1 to 4%. By comparing the leaves at 35–36th and 5–6th nodes in September (the difference in their ages is 130 days), we found that Q-glr ratio decreased from 78 to 67% while Q-glc fraction increased from 11 to 19%. Furthermore, K-glc fraction increased from 2 to 5%.

## Discussion

Since flavonols have multiple roles in plant defense they are intensively investigated compounds in several stress-related studies. Different abiotic stresses (e.g. cold, heat, drought, salt, high UV irradiance, etc.) can change their levels in plant organs^29–31^. Excessive ROS production leads to oxidative stress. Antioxidants are able to alleviate the oxidative damage by scavenging of ROS. According to our in vitro measurements, grapevine leaf flavonol glycosides are excellent ROS scavengers. ^·^OH radicals generated by UV-photolysis of H_2_O_2_, for instance, readily react with flavonol glycosides eliminating the oxidative species from the system (Figure S3). Flavonoids including flavonols are synthesized in plants via the phenylpropanoid pathway^32, 33^. A large number of MYB genes involved in the regulation of flavonoid biosynthesis pathway have already been identified and isolated in numerous plant species. While the synthesis of flavonol aglycones is well documented, very little information is available on their glycosylation and developmental regulation^34^. For the first time, we systematically examined the profile of flavonol glycosides (FLGs) in Cabernet Sauvignon leaves considering the age and position of the leaves. Our sampling design ensured to investigate the leaves with very different ages and also allowed to compare the leaves with the same age but in different seasons (Figure S4). The results showed that quercetin- and kaempferol glycosides are the main FLGs in the leaves (Fig. 1). Q-glr was found in the highest concentration revealing that among the enzymatic glycosylation the glucuronidation of quercetin was preferred (Fig. 2). In comparison with other red grapevine (*Vitis vinifera)* cultivars, Cabernet Sauvignon exhibit similar flavonol profile. Cabernet Franc, Merlot, Kékfrankos and Kadarka, for example, possessed the same FLGs with minor differences in the concentrations of the individual compounds^15^. In order to reveal how the leaf position influences the flavonol profile, grapevine leaf extracts were analyzed and compared along the cane at different leaf levels. The concentration profiles obtained show that the regulation of flavonol glycosylation can be different, even though the same aglycones take part in the reactions. We found that concentrations of Q-gal, Q-glc, K-glc and K-glr were strongly position or age-sensitive. These FLGs were found to be significantly higher concentrations in the older leaves (Tables S1 and S2). Their increasing concentration in the course of time suggests that the biosyntheses of Q-gal, Q-glc, K-glc and K-glr were continuous during the leaf aging, allowing their accumulations. In contrast, Q-rut and Q-glr have already reached the maximum levels in the young leaves, and their concentrations did not increase significantly further by aging (Tables S1 and S2). Indeed, a slight decreasing trends were observed towards the older leaves (Fig. 2). Our results proposed that the limitation of biosynthesis of Q-rut and Q-glr are faster than that of Q-gal, Q-glc, K-glc and K-glr. Parallel with the biosynthesis of Q-glr, which was limited to ~ 16,500 ppm DW, the concentrations of the position-sensitive FLGs increased towards the lower leaf levels (Fig. 2). Pearson’s correlation coefficients listed in Table 1 confirm the relations between these position-sensitive FLGs. Correlation analysis also suggests that regulation of biosynthesis of Q-rut and Q-glr can be related to each other. These observations indicate a yet undiscovered regulatory network of flavonol glycosylation in grapevine. Our study is the first to describe the correlations between the individual flavonol glycosides in grapevine leaves (Figs. 3 and S2).

Although FLGs are generally considered to be UV-screening materials and ROS scavengers^35, 36^, our measurements show that older leaves exhibit higher concentration of Q-gal, Q-glc, K-glc and K-glr. In contrast, Q-glr is already present with the highest concentrations in a 10-day leaves (Table S4), indicating the key importance of Q-glr in the defense processes of grapevine. In addition to their UV screening effect, FLGs as antioxidants also play key role in ROS neutralization^37–39^. In our previous study, it was showed that a strong photocatalytic oxidative stress can induce the decreasing of Q-glr content in the leaves^15^. This finding is in good agreement with the fact that among FLGs Q-glr represents the first line of defense^15^, explaining why this compound is already present in high concentrations in the young grapevine leaves. Moreover, combining the previous findings with the new results we can conclude that Q-glr biosynthesis is boosted mainly in the young leaves, and therefore a significant Q-glr deficiency (or its reduced level) induced by an oxidative stress can be remained in older leaves.

The similarity of the concentration profile of FLGs between 5 and 22th node indicates that initial flavonol levels in younger leaves (in June) will be determinant for the final flavonol contents in September (Fig. 2). During the leaf aging this initial level can increase if the FLGs’ synthesis is not limited (in case of Q-gal, Q-glc, K-glc and K-glr) or the plants are not exposed to severe oxidative stress. Note that ca. 90 days have elapsed between the two sampling date, and during this period, the trend observed in June was not overwritten by microclimatic conditions (Figure S5). Although numerous studies reported^6, 9, 10, 40^ that flavonols have key role in the protection of plants against high UV radiation we found that leaves possessed FLGs with higher concentration in September when the daily integrated UV radiation was much lower than in June (Figs. 2 and S5). For example, Q-glc level of the youngest leaves (last leaf positions) was ~ 1,125 and ~ 2,415 ppm DW in June and September, respectively. Despite of the same leaf age (10 days), the leaves harvested in September contained c.a. two times higher Q-glc level. By comparing the last leaf positions from June and September, further FLGs such as Q-gal, Q-glc and K-glr were found to be significantly higher level in September (Table S4). Also, by taking into consideration all the leaves along the cane, concentrations of Q-gal, Q-glc, K-glc and K-glr were significantly higher in September (Table S3). The boosted biosynthesis of these FLGs resulted in higher level of total flavonols in September (Fig. 4, Table S4). UV-B radiation is one of the most effective inducers for the biosynthesis of flavonols^6, 41, 42^. However, the increased synthesis of flavonols in September, with the reduced daily integrated UV radiation (Figure S5), indicates that besides UV light, there should be another regulatory factor in the biosynthesis of FLGs.

Due to the changes in the concentration of individual flavonols, the flavonol profile altered significantly by the age and season. In general, the aging of leaves induced the decreasing of Q-glr ratio, while Q-glc and K-glc ratios increased (Fig. 5). Taking into account that total flavonol content did not change significantly within one season, we can conclude that in older leaves, quercetin glucosidation increased while glucuronidation of quercetin reduced in favor of glucosidation. This may suggest that the regulation of the different flavonol glycosyltransferases is age-dependent in grapevine.

In summary, grapevine leaves are rich in flavonol glycosides. In turn, flavonol content and composition strongly depend on the leaf position (i.e., leaf age) and harvesting season. These factors must be strictly considered in order to ensure proper sampling when flavonols are investigated. By ignoring the leaf position and the harvesting season, can result in misleading information or misinterpretation of flavonol stress responses. Our results also highlight the need of further studies to elucidate the genetic background of flavonol glycosylation. It would be also important to clarify the environmental factors which regulate the glycosylation processes.

## Conclusions

The regulation of biosynthesis of some flavonol glycosides in Cabernet Sauvignon leaves differed significantly. We found that Q-gal, Q-glc, K-glc and K-glr formed continuously during the aging of leaves which led to the higher level of these compounds in older leaves. In contrast, Q-rut and Q-glr did not show significant accumulation in older leaves. Strong positive correlation was found between Q-rut and Q-glr levels. Furthermore, the concentration of Q-gal, Q-glc, K-glc and K-glr were also correlated suggesting a common regulation for the different glycosylation of aglycones.

Season was also important factor which influenced the level and composition of flavonols. Grapevine boosted selectively the biosynthesis of some flavonol glycosides in September relative to June. Concentration of Q-gal, Q-glc, K-glc increased considerably in September which confirmed that these compounds can form continuously by leaf aging. The flavonol profile was influenced by both age and season. The older leaves exhibited lower fraction of Q-glr and higher ratio of Q-glc and K-glr.

Our findings pointed out that leaf position and season are very important factors for sampling which should be taken into consideration when flavonol levels are studied.

## Materials and methods

### Chemicals and reagents

Acetonitrile and methanol (Promochem Optigrade, LGC Standards GmbH, Wesel, Germany) were gradient grade for liquid chromatography. Quercetin-3-*O*-rutinoside, quercetin-3-*O-*galactoside, quercetin-3-*O-*glucoside, quercetin-3-*O*-glucuronide, kaempferol-3-*O*-glucoside, and kaempferol-3-*O*-glucuronide were purchased from Extrasynthese (Genay, France). Ultrapure water system (LaboStarf 7 TWF-UV, Germany) was used to obtain high purity water.

### Experimental site and plant material

Thirteen-year-old vines of *Vitis vinifera* L. cultivar ‘Cabernet Sauvignon’ were investigated under non-irrigated open-field conditions on the south-facing slopes of Mecsek Hills, in Hungary (latitude: 46°07′ N, longitude: 18°17′ E, 200 m a.s.l.). Vines were grafted on commonly used rootstock varieties ‘T5C’ (*Vitis berlandieri* x *Vitis riparia*). The soil was a Ramann-type brown forest soil mixed with clay formed on red sandstone covered by Pannonian sediment. Vines were grown with 3.5 × 1.2 m vine spacing with East–West row direction in vertical shoot positioned umbrella training system.

Meteorological data such as natural broadband UV radiation, precipitation, temperature and relative humidity were monitored using the WS600 automatic weather station (Lufft GmbH, Germany) equipped with CUV5 radiometer (Kipp&Zonen, Delft, The Netherlands).

### Experimental design

Leaf samples at the southern side of the canopy were collected from randomly chosen shoots of nine individual vines in 14th June and 10th September 2018. Three shoots of different vines were combined and leaves were pooled at the same leaf positions. Figure S4 demonstrates the sampling design of the leaves in the two season. Since the developmental stage of shoots was strongly different in the two seasons, the shoots possessed 28 and 42 leaf levels (one leaf per node) in June and September, respectively (Figure S4). For analytical measurements, two leaves from each consecutive node were also pooled. The youngest, not fully developed leaves and basal older leaves were excluded from the sampling because of anatomical reasons. For sampling, healthy sun-exposed and fully developed leaves were collected. Based on our phenological monitoring of the cultivar Cabernet Sauvignon during the vegetation period, we set up the correlation system between leaf levels and corresponding leaf age according to BBCH scale^43–45^. The estimated leaf age with the leaf positions is displayed in Figure S4.

### Extraction of leaves

The collected grapevine leaves were immediately frozen in liquid nitrogen, then transported to the laboratory and stored at − 25 °C. The leaves were grounded in liquid nitrogen using a porcelain mortar then they were lyophilized. 25 mg of each freeze-dried sample was extracted with 1.0 ml of 60% (v/v) aqueous methanol solution acidified with formic acid (1% (v/v)), and subsequently sonicated in water bath for 15 min. The resulting suspensions were centrifuged at 20,660×*g* for 10 min. The extraction procedure was repeated three times and the combined supernatants were analysed by using HPLC–DAD system. Prior to the analysis the extracts were filtered through 0.22 μm PTFE syringe filters (Labex Ltd., Hungary).

### High-performance liquid chromatography

Chromatographic analysis was performed on a PerkinElmer Series 200 HPLC system using a Phenomenex Kinetex 2.6 μm XB-C18 100 Å, 100 × 4.6 mm column. Column temperature was maintained at 20 °C. Mobile phase with flow rate of 1 ml min^−1^ was composed of (A) 0.1% formic acid and (B) a mixture of 0.2% formic acid and acetonitrile (1:1). For the separation, the elution program was presented in Table S5. A volume of 5 μl of methanolic extract was injected into the HPLC system and the absorbance was monitored by a diode array detector at 330 nm (for caftaric acid) and 350 nm (for flavonols). Calibration curves for quantification were obtained by measuring analytical standards with known concentrations. The results were expressed in μg g^−1^ (ppm) DW.

### Statistical analysis

Statistical analyses including Pearson correlation were carried out using IBM SPSS 26.0 (IBM SPSS Inc., Chiocago, IL, USA). Pearson’s correlation coefficients were calculated to determine the degree of correlation between the individual flavonol glycosides. Standard deviation and Student's t-test were calculated on all data sets of leaf levels and components. Results were considered statistically significant at *p* < 0.05. Linear regressions on data rows were performed by using OriginPro 8.6.0.

## Supplementary information

Supplementary Information.
