# Supplementary material for: Age- and season-dependent pattern of flavonol glycosides in Cabernet Sauvignon grapevine leaves
Source: Sci Rep. 2020 Aug 28;10:14241. doi: 10.1038/s41598-020-70706-7 (PMC7455724; doi:10.1038/s41598-020-70706-7)
Supplement: Supplementary file 1 — Supplementary Information. [file 41598_2020_70706_MOESM1_ESM.docx]

**Age- and season-dependent pattern of flavonol glycosides in Cabernet sauvignon grapevine leaves**

Sakina Bouderias ^1,2^, Péter Teszlák ^1^, Gábor Jakab ^1,2^ László Kőrösi ^1,*^

^1^ Research Institute for Viticulture and Oenology, University of Pécs, Pázmány P. u. 4, Pécs H-7634, Hungary

^2^ Department of Plant Biology, University of Pécs, Ifjúság u. 6, Pécs H-7624, Hungary

* Corresponding author. E-mail: korosi.laszlo@pte.hu;

**SUPPLEMENTARY MATERIAL**

**Figure S1.** Chemical structures of the identified flavonol glycosides in Cabernet Sauvignon leaves


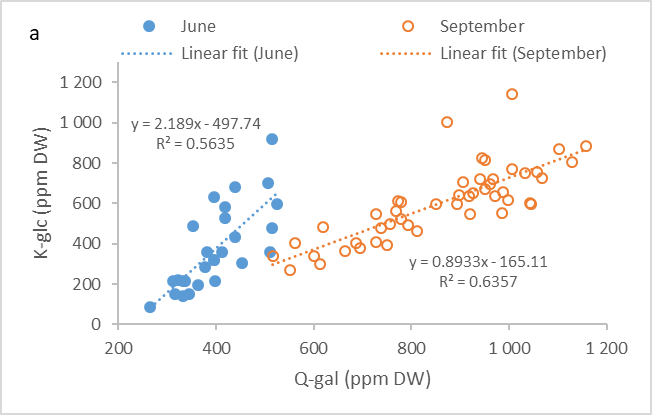

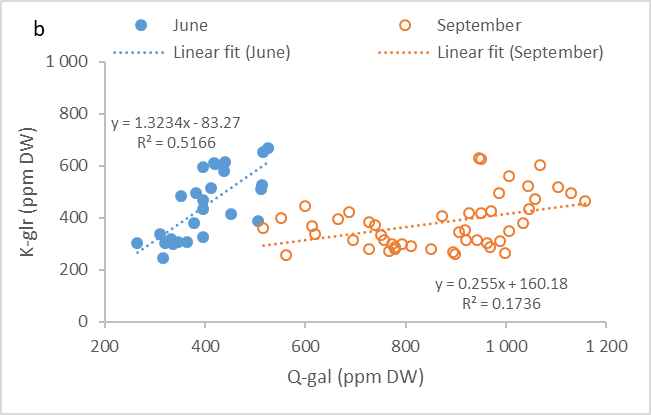

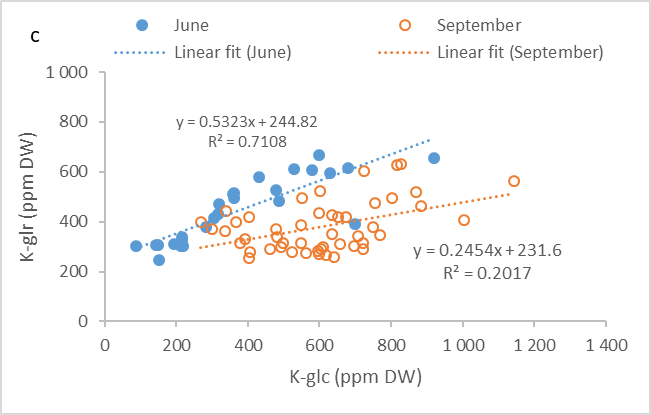

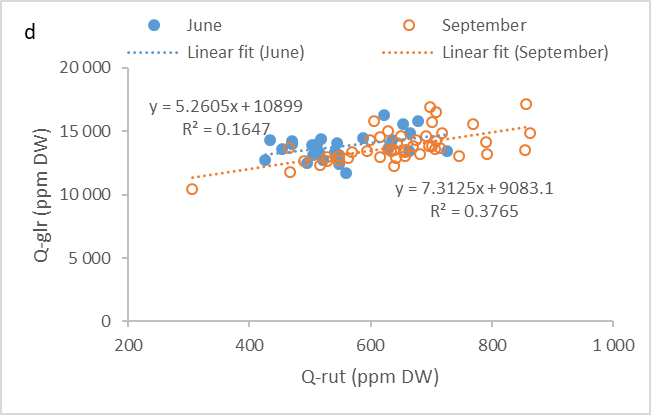


**Figure S2.** Plotting of (a) K-glc vs. Q-gal, (b) K-glr vs. Q-gal, (c) K-glr vs. K-glc and (d) Q-glr vs. Q-rut.

**Figure S3.** Monitoring of flavonol composition of an aqueous grapevine leaf extract containing 140 mmol/L H_2_O_2_ under UV-A irradiation as a function of time.

Based on the following equation, ^●^OH radicals were generated by UV-photolysis of H_2_O_2_: H_2_O_2_ + *hν* → ^●^OH + ^●^OH

Due to the scavenging of ^●^OH radicals, the level of flavonol glycosides decreased gradually as a function of time. The concentration of quercetin-3-*O*-rutinoside (Q-rut), quercetin-3-*O*-galactoside (Q-gal), quercetin-3-*O*-glucoside (Q-glc), kaempferol-3-*O*-glucoside (K-glc), kaempferol-3-*O*-glucuronide (K-glr) are depicted on the left axis of the diagram while the concentration of quercetin-3-*O*-glucuronide (Q-glr) is presented on the right axis.

**Figure S4.** Schematic draw of Cabernet Sauvignon shoots with 28 and 42 leaf levels harvested in June and September, respectively. Black numbers demonstrate the leaf levels while blue numbers on the right side show the corresponding leaf age. The green marks indicate which leaves were analysed by HPLC-DAD.

**Figure S5.** Meteorological data between April and September in 2018: (a) mean temperature, (b) daily integrated UV-A and B radiation, (c) amount of precipitation and (d) relative humidity of air. In 2018, the average annual temperature was 14.1 °C, the site receives 717 mm annual precipitation and 2186 hours of sunshine.

**
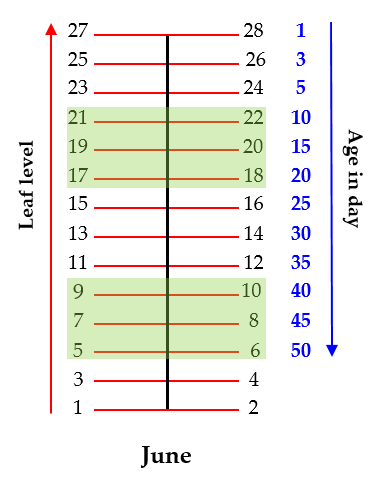
Table S1.** Flavonol levels and total flavonol content of the leaves harvested from 5-10 and 17-22 leaf levels in June. Data are expressed in μg g^-1^ dry weight. Results clearly demonstrate that the amounts of Q-gal, Q-glc, K-glc and K-glr were significantly lower at higher leaf levels (i.e. younger leaves). Q-rut, Q-glr and total flavonol concentrations did not differ significantly between the two age-groups.

|  | | | Q-rut | Q-gal | Q-glc | Q-glr | K-glc | K-glr | Total flavonol |
| --- | --- | --- | --- | --- | --- | --- | --- | --- | --- |
| June | Leaves  17-22 | Shoot#1 | 616±65 | 305±35 | 1066±171 | 14831±2088 | 128±35 | 285±35 | 17231±2046 |
|  |  | Shoot#2 | 610±86 | 360±32 | 1349±141 | 14814±828 | 193±37 | 311±13 | 17637±1035 |
|  |  | Shoot#3 | 466±47 | 322±11 | 1269±23 | 13565±844 | 216±3 | 321±19 | 16159±908 |
|  |  | mean±sd^a^ | 564±85 | 329±28 | 1228±146 | 14403±726 | 179±46 | 306±18 | 17009±764 |
|  |  | |  |  |  |  |  |  |  |
|  | Leaves  5-10 | Shoot#1 | 613±64 | 493±47 | 2014±176 | 13702±525 | 586±102 | 603±71 | 18011±779 |
|  |  | Shoot#2 | 522±20 | 443±63 | 1884±342 | 13510±525 | 693±203 | 621±30 | 17672±1165 |
|  |  | Shoot#3 | 458±21 | 426±77 | 1926±434 | 14180±150 | 589±106 | 494±110 | 18074±429 |
|  |  | mean±sd^a^ | 531±78 | 454±35 | 1941±67 | 13797±345 | 623±61 | 573±69 | 17919±216 |
|  |  | p (t-test)^b^ | 0.6437 | 0.0083 | 0.0015 | 0.2615 | 0.0005 | 0.0029 | 0.1181 |

^a^Mean values ± standard deviation for the individual shoots (1-3) with 5-10 or 17-22 leaf levels.

^b^P is the results of t-test between the 5-10 and 17-22 leaves.

**Table S2.** Flavonol levels and total flavonol content of the leaves harvested from 5-10^th^ and 31-36 ^th^ leaf levels in September. Data are expressed in μg g^-1^ dry weight. Q-gal, Q-glc, K-glc and K-glr significantly lower for younger leaves (at 31-36^th^ leaf levels). Q-rut, and total flavonol concentrations did not differ significantly between the two age-groups. Q-glr concentration significantly increased in the younger leaves.


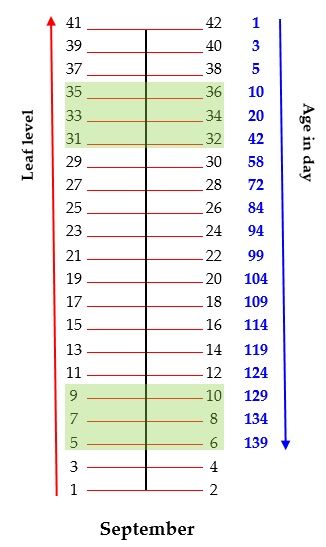


|  | | | Q-rut | Q-gal | Q-glc | Q-glr | K-glc | K-glr | Total flavonol |
| --- | --- | --- | --- | --- | --- | --- | --- | --- | --- |
| September | Leaves  31-36 | Shoot#1 | 687±85 | 782±59 | 3194±392 | 14830±652 | 524±62 | 323±46 | 20340±412 |
|  |  | Shoot#2 | 723±126 | 562±53 | 2205±343 | 16480±668 | 362±108 | 367±30 | 20699±408 |
|  |  | Shoot#3 | 671±49 | 679±58 | 2746±325 | 15706±1186 | 362±55 | 321±45 | 20484±846 |
|  |  | mean±sd^a^ | 694±27 | 674±110 | 2715±496 | 15672±826 | 416±94 | 337±26 | 20508±180 |
|  |  | |  |  |  |  |  |  |  |
|  | Leaves  5-10 | Shoot#1 | 472±168 | 884±237 | 3528±1309 | 12091±1817 | 665±191 | 425±55 | 18015±3747 |
|  |  | Shoot#2 | 752±38 | 999±50 | 3976±409 | 13681±1013 | 857±273 | 572±54 | 20836±1614 |
|  |  | Shoot#3 | 641±150 | 964±98 | 3717±433 | 13217±810 | 848±142 | 547±121 | 19934±1390 |
|  |  | mean±sd^a^ | 622±141 | 949±59 | 3741±225 | 12996±818 | 790±108 | 514±79 | 19595±1441 |
|  |  | p (t-test)^b^ | 0.4339 | 0.0188 | 0.0310 | 0.0163 | 0.0106 | 0.0205 | 0.3396 |

**
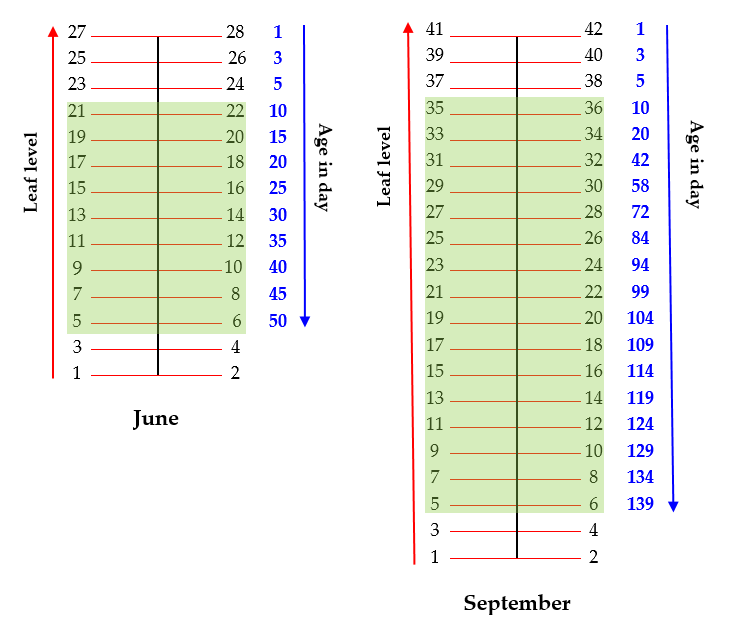
Table S3.** Flavonol levels and total flavonol content of the leaves harvested in June and September. Leaves were collected from 5-22^th^ and 5-36^th^ leaf levels in June and September, respectively.

|  | Leaves  5-36 | Q-rut | Q-gal | Q-glc | Q-glr | K-glc | K-glr | Total flavonol |
| --- | --- | --- | --- | --- | --- | --- | --- | --- |
| September | Shoot#1 | 616±109 | 942±141 | 3949±733 | 13711±1228 | 678±125 | 369±84 | 20265±1899 |
|  | Shoot#2 | 660±119 | 832±182 | 3433±780 | 13860±1428 | 611±203 | 389±106 | 19785±1274 |
|  | Shoot#3 | 656±92 | 815±146 | 3310±623 | 13810±1240 | 528±193 | 383±114 | 19501±1387 |
|  | mean±sd^a^ | 644±24 | 863±69 | 3564±339 | 13793±76 | 605±75 | 380±10 | 19850±386 |
|  | Leaves  5-22 |  |  |  |  |  |  |  |
| June | Shoot#1 | 626±60 | 413±97 | 1591±459 | 14149±1224 | 333±213 | 434±153 | 17547±1152 |
|  | Shoot#2 | 557±61 | 396±51 | 1584±301 | 13679±1126 | 402±248 | 456±139 | 17075±1241 |
|  | Shoot#3 | 477±35 | 388±64 | 1623±366 | 13601±755 | 392±173 | 445±113 | 16927±1135 |
|  | mean±sd^a^ | 553±74 | 399±13 | 1600±21 | 13810±297 | 376±37 | 445±11 | 17183±324 |
|  | p (t-test)^b^ | 0.11475 | 0.0003 | 0.0104 | 0.9303 | 0.0090 | 0.0018 | 0.0008 |

^a^Mean values of flavonols for all the leaves of the individual shoots (#1-#3). Data are expressed in μg g^-1^ dry weight.

^b^P values show the results of t-test between 5-22^th^ and 5-36^th^ leaf levels.


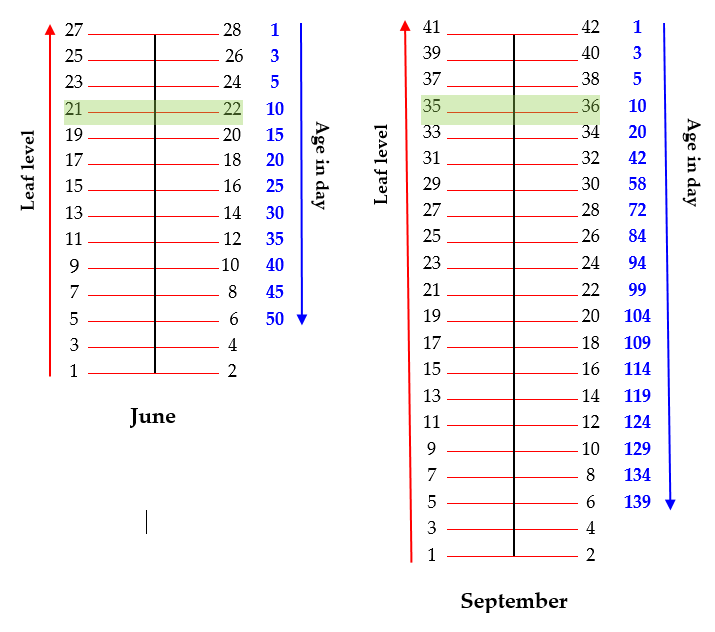
**Table S4.** Comparison the leaves with same age but in different seasons. 10-days-leaves leaves harvested from 21-22^th^ and 35-36 ^th^ leaf levels in June and September, respectively. Flavonol levels and total flavonol content are expressed in μg g^-1^ dry weight. Q-gal, Q-glc, and K-glr and total flavonol levels were significantly (P<0.05) higher in September. Q-rut, Q-glr and K-glc concentrations did not differ significantly between the two seasons.

|  | | | Q-rut | Q-gal | Q-glc | Q-glr | K-glc | K-glr | Total flavonol |
| --- | --- | --- | --- | --- | --- | --- | --- | --- | --- |
| September | Leaves  35-36 | Shoot#1 | 769 | 739 | 2865 | 15561 | 480 | 372 | 20786 |
|  |  | Shoot#2 | 856 | 551 | 1982 | 17111 | 268 | 399 | 21168 |
|  |  | Shoot#3 | 698 | 614 | 2397 | 16894 | 300 | 369 | 21272 |
|  |  | mean±sd^a^ | 774±79 | 635±96 | 2415±442 | 16522±839 | 349±114 | 380±17 | 21075±256 |
|  |  | |  |  |  |  |  |  |  |
| June | Leaves  21-22 | Shoot#1 | 622 | 265 | 868 | 16285 | 88 | 303 | 18431 |
|  |  | Shoot#2 | 666 | 345 | 1226 | 14874 | 151 | 307 | 17567 |
|  |  | Shoot#3 | 518 | 333 | 1280 | 14401 | 214 | 320 | 17066 |
|  |  | mean±sd^a^ | 602±76 | 315±43 | 1125±224 | 15187±980 | 151±63 | 310±9 | 17688±691 |
|  |  | p (t-test)^b^ | 0.0529 | 0.0062 | 0.0107 | 0.1475 | 0.0573 | 0.0029 | 0.0013 |

**Table S5.** Elution profile for HPLC-DAD analysis

| Step | time (min) | A(%) | B(%) | Curve |
| --- | --- | --- | --- | --- |
| 0 | 15 | 100 | 0 | 0 |
| 1 | 5 | 100 | 0 | 0 |
| 2 | 3 | 90 | 10 | 0 |
| 3 | 5 | 80 | 20 | -9 |
| 4 | 8 | 60 | 40 | -2 |
| 5 | 4 | 30 | 70 | 1 |
| 6 | 3 | 0 | 100 | 0 |
